# Thermoelectric Properties of Ba_2–*x*_Eu_*x*_ZnSb_2_, a Zintl Phase with One-Dimensional Covalent Chains

**DOI:** 10.1021/acs.inorgchem.2c04484

**Published:** 2023-04-06

**Authors:** Ashlee K. Hauble, Kamil Ciesielski, Valentin Taufour, Eric S. Toberer, Susan M. Kauzlarich

**Affiliations:** † Department of Chemistry, University of California, One Shields Avenue, Davis, California 95616, United States; ‡ Department of Physics, Colorado School of Mines, 1500 Illinois Street, Golden, Colorado 80401, United States; § Department of Physics and Astronomy, University of California, One Shields Avenue, Davis, California 95616, United States

## Abstract

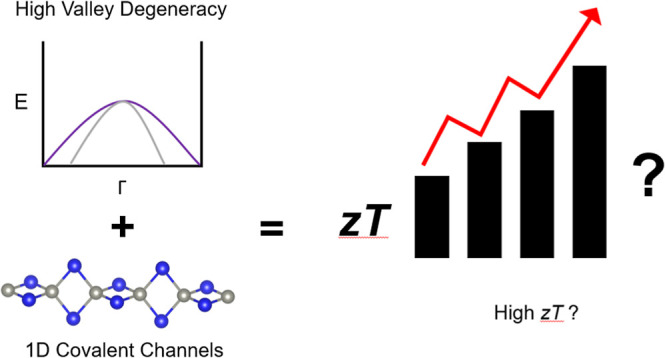

The compound Ba_2_ZnSb_2_ has been predicted to be a promising thermoelectric material, potentially achieving *zT* > 2 at 900 K due to its one-dimensional chains of edge-shared [ZnSb_4/2_]^4–^ tetrahedra and interspersed Ba cations. However, the high air sensitivity of this material makes it difficult to measure its thermoelectric properties. In this work, isovalent substitution of Eu for Ba was carried out to make Ba_2–*x*_Eu_*x*_ZnSb_2_ in order to improve the stability of the material in air and to allow characterization of thermal and electronic properties of three different compositions (*x* = 0.2, 0.3, and 0.4). Polycrystalline samples were synthesized using binary precursors via ball milling and annealing, and their thermoelectric properties were measured. Samples showed low thermal conductivity (<0.8 W/m K), a high Seebeck coefficient (350–550 μV/K), and high charge carrier mobility (20–35 cm^2^/V) from 300 to 500 K, consistent with predictions of high thermoelectric efficiency. Evaluation of the thermoelectric quality factor suggests that a higher *zT* can be attained if the carrier concentration can be increased via doping.

## Abstract 2

Ba_2_ZnSb_2_ is a Zintl phase with one-dimensional chains and high valley degeneracy at the valence band maximum. The anisotropic bonding motif provides bands that contribute light and heavy effective mass, positively impacting both the Seebeck coefficient and electrical resistivity. Polycrystalline Ba_2−*x*_Eu_*x*_ZnSb_2_ synthesized from binary precursors shows low thermal conductivity and high Seebeck coefficients. Further optimization of carrier concentration will improve the electrical resistivity and may lead to the predicted high thermoelectric efficiency.

## 1 Introduction

Thermoelectric generators have the potential to alter the world’s energy landscape by converting heat to usable electricity, which could reduce greenhouse gas emissions and dependence on fossil fuels. To make this possible, materials with a high thermoelectric figure of merit, *zT* = (*S*^2^*T*)/κρ (where *S* is the Seebeck coefficient, *T* is the operating temperature, κ is thermal conductivity, and ρ is electrical resistivity), need to be designed. Zintl phases are a class of intermetallic compounds that is well suited to this application, given the rich structural chemistry that can result from the combination of ionic and covalent bonding found in these materials.^1^ The primary challenge in designing a thermoelectric material is to simultaneously improve these three properties, a task that is difficult due to the interrelated nature of *S*, ρ, and the electronic component of κ.^2−4^

One method of optimizing both the Seebeck coefficient (*S*) and resistivity (ρ) simultaneously is to look for materials containing anisotropic bonding schemes that provide a favorable Seebeck coefficient in one crystallographic direction and resistivity in another. Some anisotropic layered and low-dimensional materials are promising candidates for thermoelectric applications because they decouple the Seebeck and electrical resistivity effective masses, with the electrical resistivity being governed by the light mass in one crystallographic direction while the Seebeck coefficient is controlled by the average mass.^5−8^ Examples of this strategy include layered materials with A_2_MPn_2_ stoichiometry and materials containing 1D tetrahedral chains, such as the A_3_MPn_3_ and A_5_M_2_Pn_6_ families of compounds, which have demonstrated competitive *zT*s due to their high charge carrier mobilities and Seebeck coefficients.^9−17^

Ba_2_ZnSb_2_ is a Zintl phase with infinite chains of 1D edge-shared [ZnSb_4/2_]^4–^ tetrahedra that are structurally similar to the corner-shared infinite tetrahedral chains in Ca_5_Al_2_Sb_6_ compounds, the discrete corner-shared chains in Ca_3_AlSb_3_, and the isolated edge-shared chains in Sr_3_AlSb_3_.^3,18,19^ These covalent chains can form channels that transport charge and result in high mobility, which is good for thermoelectric performance. Motivated by these structural motifs, theoretical investigations of the thermoelectric properties and electronic structure of p-type Ba_2_ZnSb_2_ revealed an anisotropic Fermi surface with high valley degeneracy (*N*_v_ = 4), low lattice thermal conductivity, and predicted an average *zT* > 1, with *zT* > 2 in the *z*-direction at 900 K.^5,18,20^ The two bands at the edge of the valence band are flat and heavy, which give rise to a high Seebeck coefficient, while the two that are just below are lighter bands that are expected to result in high charge carrier mobility and low electrical resistivity.^5,18^

The synthesis, characterization, and experimental thermoelectric properties for Ba_2–*x*_Eu_*x*_ZnSb_2_ (*x* = 0.2, 0.3, and 0.4) are reported. Small amounts of Eu were substituted for Ba to reduce air sensitivity enough to make measurements feasible while retaining the orthorhombic structure type of Ba_2_ZnSb_2_ and avoiding a phase transition to the hexagonal Eu_2_ZnSb_2_ structure type.

## 2 Methods

### 2.1 Synthesis

Samples were synthesized by ball milling elements with binary precursors and annealing the resultant powder. Ba_11_Sb_10_ and Eu_11_Sb_10_ were used as cation sources to eliminate difficulties associated with milling highly malleable and reactive alkaline-earth and rare earth metals.^21^ A_11_Sb_10_ precursors were also synthesized via high-energy ball milling and annealing. In an Ar-filled glovebox, pea-sized pieces were clipped off a Ba rod (Sigma-Aldrich, 99.999%), Eu pieces (Stanford materials, 99.99%) were cut to a similar size, and each was combined with the Sb shot (5N Plus, 99.999%) that had been ground into a fine powder using an agate mortar and pestle. Both reactions were sealed in a ball mill vial, as described above, and milled. After 30 min, both reactions were brought into the glovebox to be scraped, resealed, and then milled for an additional 30 min. The milled powders were sealed into tubes (Nb for Ba and Ta for Eu) and jacketed in evacuated quartz to be annealed (200 °C/h to 800 °C, dwell for 12 h). The diffraction patterns are provided in Supporting Information, Figure S1, with Rietveld refinement parameters (lattice, % phases, and statistics) in Table S1.

Due to the air sensitivity of Ba_2–*x*_Eu_*x*_ZnSb_2_, all manipulations were carried out in an Ar-filled glovebox. The synthesized A_11_Sb_10_ (A = Ba, Eu) precursors were combined with elemental Zn flakes (Alfa, 99.98%) and a small amount of Sb shot (5N Plus, 99.999%) in a 65 mL stainless-steel ball mill vial with two 12.7 mm diameter stainless-steel balls, hermetically sealed in a metalized 4 mil polyethylene bag and milled in a SPEX 8000 M mill for 30 min, then scraped with a stainless-steel spatula, resealed, and milled for an additional 30 min. The homogenized powder was then scraped from the container, and approximately 3 g was packed into a 7 cm long, 1 cm diameter Nb tube that was welded shut in an argon arc welder and flame sealed in an evacuated quartz jacket (<50 mTorr) to be annealed in a box furnace. The reaction was heated at a rate of 200 °C/h to 800 °C and dwelled for 96 h. Samples of Ba_2–*x*_Eu_*x*_ZnSb_2_ with Eu content *x* = 0, 0.2, 0.3, and 0.4 were successfully synthesized as determined by powder X-ray diffraction (Rietveld refinement provided in Supporting Information, Figures S2 and S3, with refinement parameters (lattice parameters, % phases, and statistics in Table S2). Property measurements were not carried out for *x* = 0 due to air sensitivity. Attempts were made to increase Eu content, but products of *x* = 0.5 and 0.75 provided a combination of the orthorhombic Ba_2_ZnSb_2_ structure type and the hexagonal Eu_2_ZnSb_2_ structure type, while *x* = 1 gave only the Eu_2_ZnSb_2_ structure type. As this study is focused on the Ba_2_ZnSb_2_ structure type, characterization, and properties, they are presented only for *x* = 0.2, 0.3, and 0.4.

### 2.2 Spark Plasma Sintering

In a glovebox, the annealed powders of Ba_2–*x*_Eu_*x*_ZnSb_2_*x* = 0.2, 0.3, and 0.4 were ground with an agate mortar and pestle, sieved (100 mesh), and packed into a graphite die (12.7 mm inner diameter) that had been lined with graphite foil. The die was loaded into the chamber of a Dr. Sinter Jr. instrument (Fuji Electronic Industrial Co., Ltd.), which was evacuated below 15 Pa, refilled to 50,000 Pa of Ar, and sintered according to the following profile: heated to 600 °C in 15 min and then to 650 °C in 1 min (to prevent temperature overshoot) and dwelled at 650 °C for 20 min. At 400 °C, the applied pressure was slowly increased from 47 to 83 MPa. The geometric densities of the consolidated pellets were >95% of the theoretical values. Samples were polished with dried sandpaper inside an argon-filled glovebox to prevent oxidation.

### 2.3 Air-free Powder X-ray Diffraction (PXRD)

Portions of the pressed pellets were prepared for air-free PXRD characterization by grinding with an agate mortar and pestle, sieving the powder onto double-sided polyimide tape on a zero background PXRD plate, and protecting from air with a polyimide film in an Ar-filled glovebox. A Bruker D8 ADVANCE Eco diffractometer with Cu Kα radiation operating at 40 kV and 25 mA was used to collect diffraction data from the 2θ range of 15–60°. The step size was 0.015, and the scan rate was 1 s per step. TOPAS5 software was used for Rietveld refinement of the data to refine lattice parameters and obtain information concerning phase purity. Rietveld refinements of binary precursors and Ba_2–*x*_Eu_*x*_ZnSb_2_ (*x* = 0, 0.2, 0.3, and 0.4) are presented in Supporting Information, Figures S1–S3 and Tables S1 and S2.

### 2.4 Scanning Electron Microscopy (SEM) and Energy-Dispersive X-ray Spectroscopy (EDS)

Slices of the consolidated pellets were mounted in epoxy pucks and polished using dried sandpaper (up to 1200 grit) inside a glovebox and loaded into a Thermo Fisher Quattro ESEM. An Everhart–Thornley detector was used to collect secondary electron micrographs with a 20 kV accelerating voltage, and an annular backscattered detector was used for Z-contrast micrographs. A Bruker Quantax EDX detector was used to determine stoichiometry and collect elemental maps.

### 2.5 Magnetism

Magnetization measurements of Ba_2–*x*_Eu_*x*_ZnSb_2_*x* = 0.2, 0.3, and 0.4 were performed in a Quantum Design magnetic property measurement system (MPMS). A polycrystalline sample (typical mass 50 mg) was placed in between two plastic straws, which was then attached to the sample holder rod and field-cooled (FC) from room temperature to 2 K. A magnetic field of 0.1 T was applied, and data were collected from 2 to 300 K (ZFC) and from 300 to 2 K (FC). A modified Curie Weiss fit was used to determine the Eu^2+^ content, assuming the theoretical value of the effective moment for Eu^2+^ ions.

### 2.6 Thermoelectric Properties

A custom-built instrument was used for Seebeck coefficient measurements from 300 to 600 K in a low-pressure (300 Torr) N_2_ atmosphere.^22^ Graphite foil was used between the sample and thermocouples to ensure good electrical contact. Although Ba_2–*x*_Eu_*x*_ZnSb_2_*x* = 0.2, 0.3, and 0.4 samples were determined to be stable up to 783 °C via differential scanning calorimetry, the upper temperature of measurements was determined based on sample behavior with repeated heating and cooling cycles. Above 550 K, the materials seem to react with the small amount of oxygen present in the atmosphere of the device. Resistivity and Hall effect measurements were carried out on a home-built system with van der Pauw geometry.^23^ A magnetic field of 1 T and a current of 0.1 A were used for measurement. A voltage–current curve was generated before measurement to confirm Ohmic contacts. Thermal diffusivity was measured using a Netzsch Laser Flash Analysis (LFA) 475 Microflash instrument, and thermal conductivity was calculated according to the equation κ = λρ*C*_p_, where κ is the thermal conductivity, *C*_p_ is the Dulong–Petit heat capacity, λ is the thermal diffusivity, and ρ is the sample density. Third-order polynomial fits of the data are presented herein and employed to calculate *zT*. The experimental data with the polynomial fits are provided in Supporting Information, Figures S4 and S5.

## 3 Results and Discussion

### 3.1 Sample Purity and Composition

**Figure 1:**
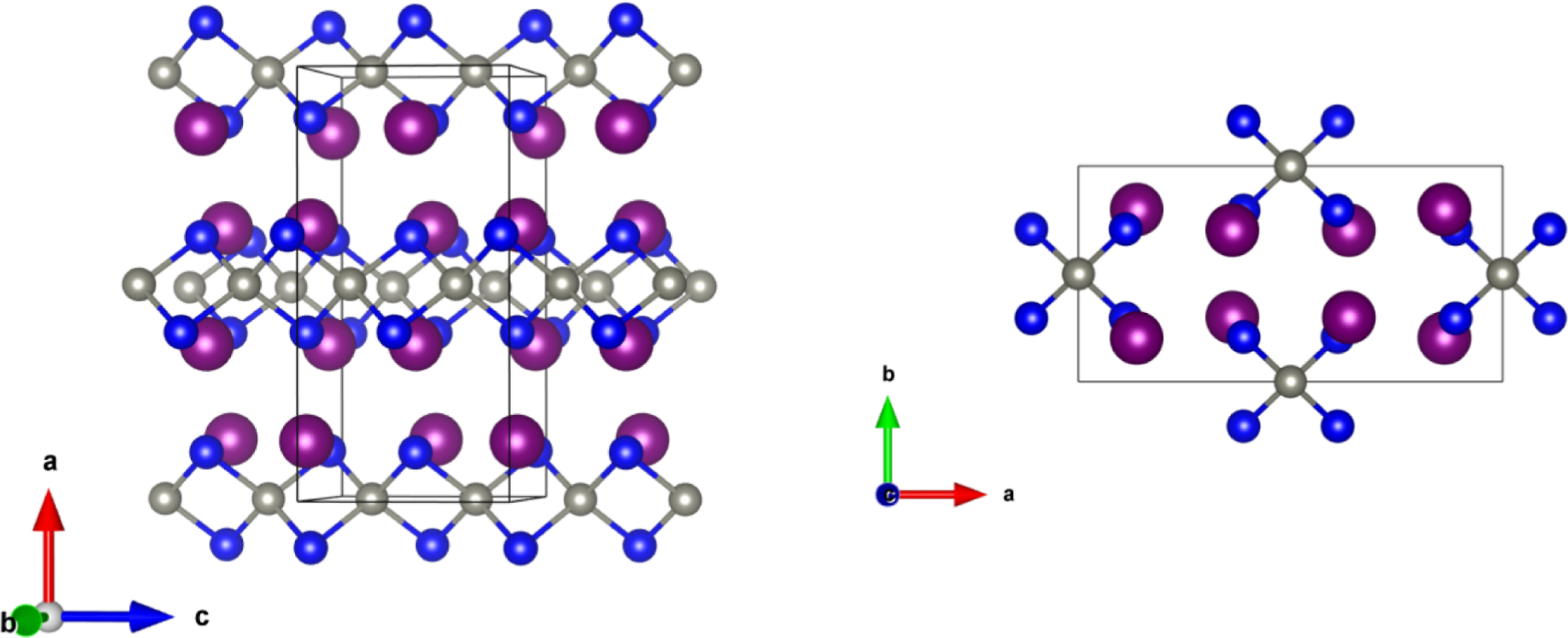
Ba_2_ZnSb_2_ structure viewed along the *b*-axis (left) and *c*-axis (right). Ba, Zn, and Sb are indicated by purple, gray, and blue colored spheres, respectively.

Figure 1 depicts the crystal structure of Ba_2_ZnSb_2_. It is a Zintl phase with three crystallographically unique sites corresponding to each atom: Ba, Zn, and Sb. Ba cations are separated by isolated infinite chains of edge-shared 1D  tetrahedra.^18,19^ These chains run along the *c* direction and are stacked in the *b* direction. The Zintl formalism can be used to rationalize the charge balance as (Ba^2+^)_2_(Zn^2+^)(Sb^3–^)_2_. Ba_2_ZnSb_2_ adopts the *Ibam* space group and has the K_2_SiP_2_ structure type.

**Figure 2:**
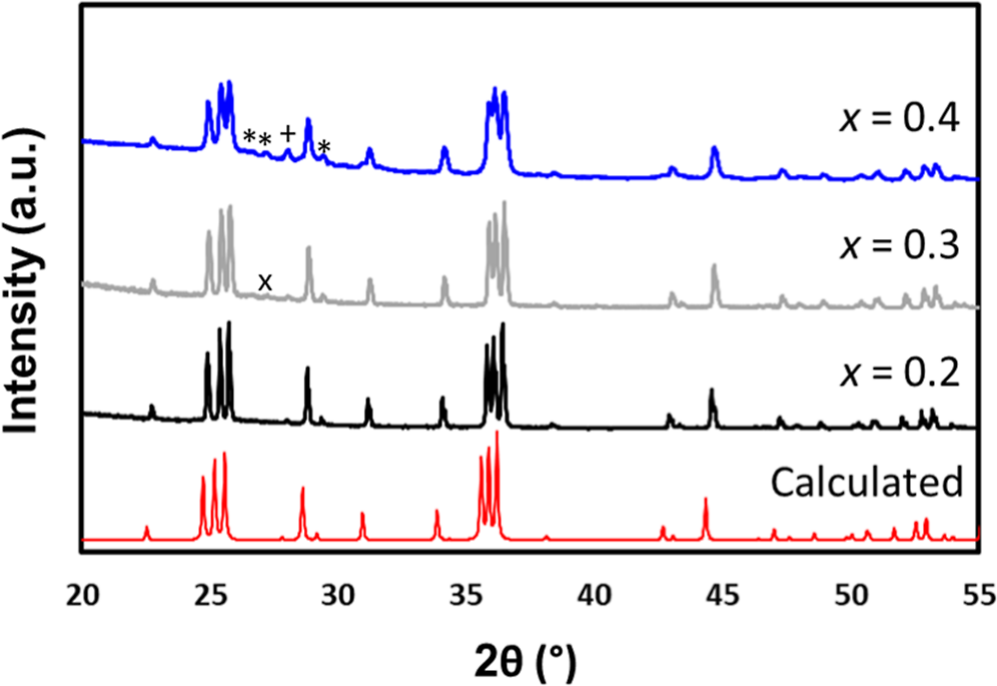
PXRD patterns for Ba_2–*x*_Eu_*x*_ZnSb_2_ (*x* = 0.2, 0.3, and 0.4) compared to the calculated pattern from the single-crystal CIF^19^ on the bottom. BaZn_2_Sb_2_ impurities are marked by asterisks, and BaO is marked by +. An unidentified peak in *x* = 0.3 is indicated with an *x*.

**Figure 3:**
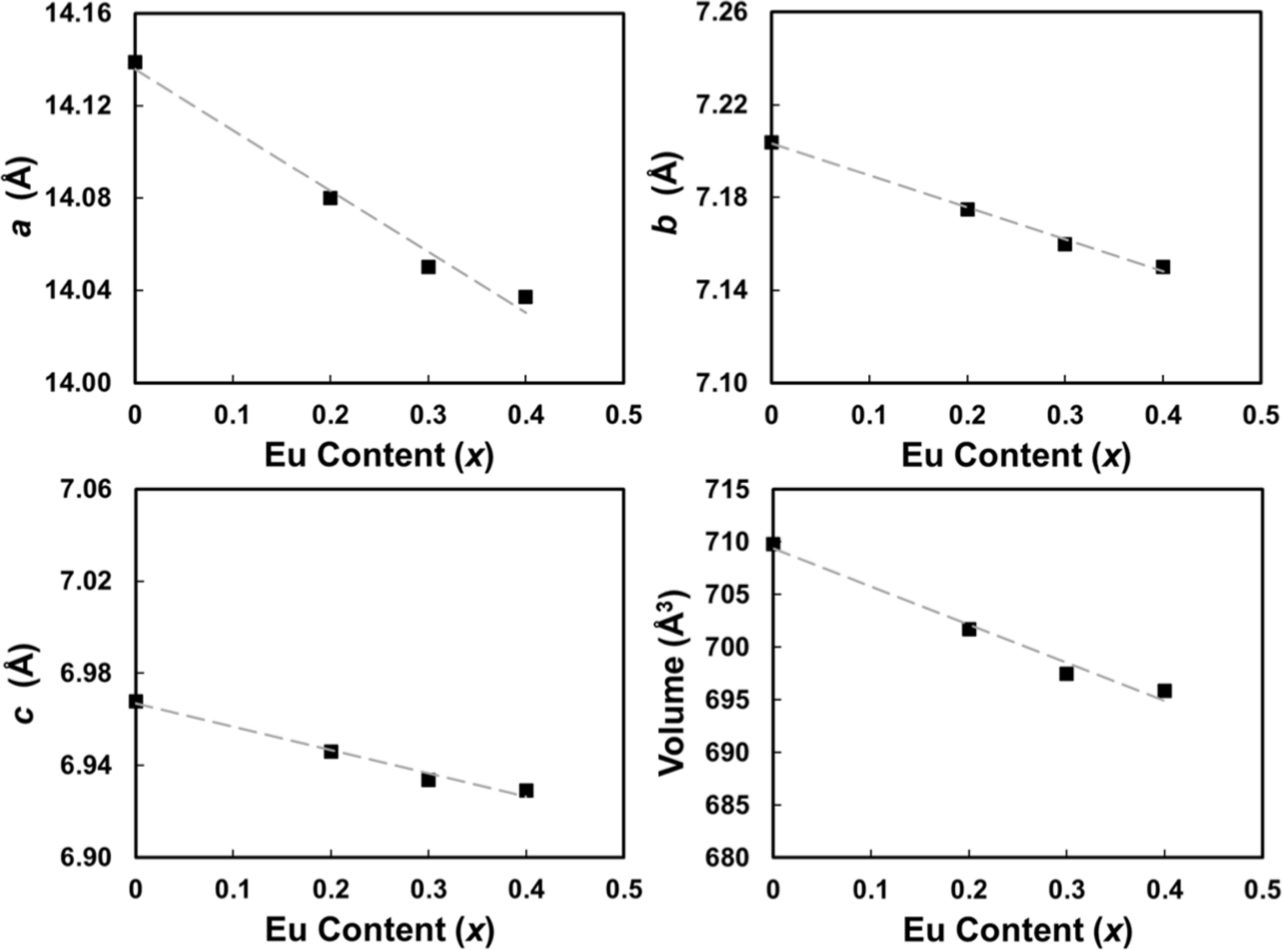
Lattice parameters as a function of Eu content for Ba_2–*x*_Eu_*x*_ZnSb_2_ (*x* = 0, 0.2, 0.3, and 0.4) from Rietveld refinement.

In the case of the solid solution of Ba_2–*x*_Eu_*x*_ZnSb_2_, Eu^2+^ is expected to substitute on the Ba^2+^ site, and because it is less electropositive than Ba^2+^, it reduces the air-sensitivity of the compound. PXRD patterns for Ba_2–*x*_Eu_*x*_ZnSb_2_ (*x* = 0.2, 0.3, and 0.4) are shown in Figure 2 and compared to the calculated pattern.^19^ Rietveld refinements employing the Ba_2_ZnSb_2_ CIF^19^ to obtain lattice parameters and possible secondary phases are provided in Supporting Information, Figures S2 and S3, and Table S2. The *x* = 0.2 sample was confirmed phase pure via Rietveld refinements, and *x* = 0.3 contained one small peak at 2θ ∼ 27° that is consistent with BaZn_2_Sb_2_ (marked by an *x* in Figures 2 and S2) but could not be indexed via Rietveld refinement since no other peaks were present, and *x* = 0.4 had <4% of a BaZn_2_Sb_2_ impurity marked by asterisks and ∼0.5% of BaO impurity marked by a plus sign. Rietveld refinements yielded *R*_wp_ statistics <10%. Lattice parameters (Figure 3, Supporting Information Table S2) decrease with *x*, as expected due to the smaller size of Eu compared to Ba. Standard deviation values for lattice parameters are smaller than the symbols used and are not shown in Figure 3.

**Figure 4:**
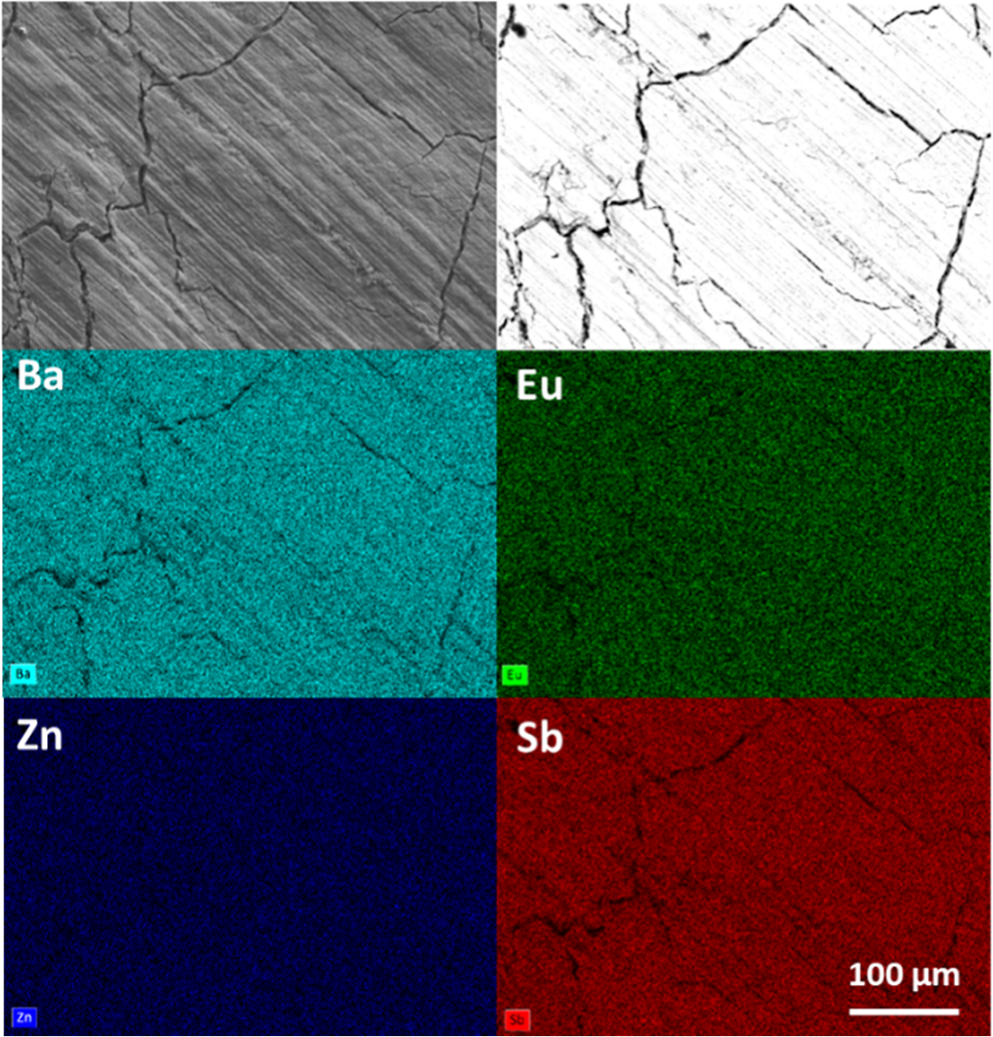
Secondary electron micrograph (top left), backscattered electron micrograph (top right), and elemental maps for the Ba_2–*x*_Eu_*x*_ZnSb_2_*x* = 0.4 pellet with Ba in turquoise, Eu in green, Zn in dark blue, and Sb in red (bottom).

**Figure 5:**
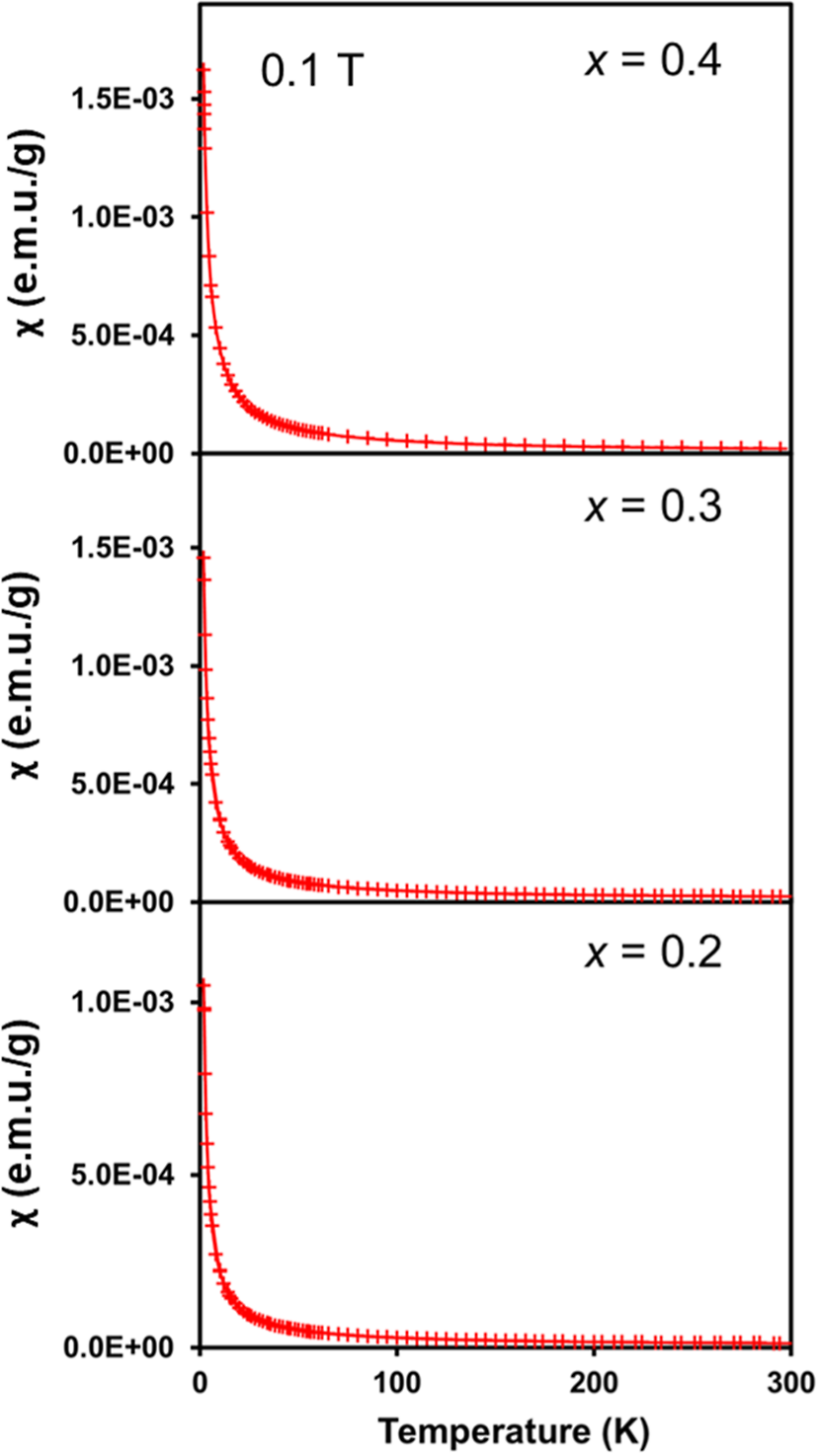
Temperature-dependent magnetization versus temperature for Ba_2–*x*_Eu_*x*_ZnSb_2_ (*x* = 0.2, 0.3, and 0.4). A line is shown to guide the eye. The three samples are plotted as M vs T in one graph, provided in Supporting Information, Figure S9.

A typical backscattered electron micrograph with elemental maps of a pressed pellet of Ba_2–*x*_Eu_*x*_ZnSb_2_*x* = 0.4 is shown in Figure 4. Elemental maps confirm the uniform distribution of all elements, and backscattered electron micrographs show that all samples are single-phase (Supporting Information, Figure S6). The cracks shown in the sample topography are due to surface oxidation that occurred during sample preparation. EDS characterization (Table 1, Supporting Information, Figure S7) confirms Eu incorporation consistent with the nominal value for *x* = 0.2 and 0.3 and *x* = 0.4. There are many examples of Eu substituting for an alkaline earth-containing Zintl phases.^24−26^ Typically, Eu substitutes into alkaline earth-containing compounds as Eu^2+^.^27−29^ Therefore, temperature-dependent magnetic susceptibility (Figure 5, Supporting Information, Figure S8) was employed to further confirm the Eu content. The magnetization as a function of temperature of the samples follows a modified Curie–Weiss behavior with a small Weiss constant and no notable magnetic ordering down to 2 K. It is expected that Eu should be Eu^2+^, and if we assume that is the case, then the *x* values obtained are 0.177(1), 0.277(1), and 0.385(2), consistent with the amounts of Eu employed in the reaction. The data plotted as inverse magnetization vs temperature showing the fits are provided in Supporting Information, Figure S9. Attempts made to incorporate more Eu (*x* = 0.5, 0.75, and 1) resulted in a mixture of the Ba_2_ZnSb_2_ and Eu_2_ZnSb_2_ structure types,^19,24^ where lattice parameters for the Ba_2_ZnSb_2_ phase matched those determined for the *x* = 0.4 sample. This result combined with the small BaZn_2_Sb_2_ impurity and slightly larger lattice parameters than expected suggests that slightly less than *x* = 0.4 is the upper limit of Eu solubility in this system.

**Table 1:** Eu Composition Determined by EDS and Magnetic Data for Ba_2–*x*_Eu_*x*_ZnSb_2_

loaded composition (*x*)	experimental *x* (EDS)	experimental *x* (magnetism)	μ (μ_Β_)^a^	θ_CW_ (K)	χ_0_ emu/g
0.2	0.20(2)	0.177(1)	7.94	–0.9(2)	4.76(5) × 10^–6^
0.3	0.29(2)	0.277(1)	7.94	–1.0(2)	1.17(1) × 10^–5^
0.4	0.37(4)	0.385(1)	7.94	–1.8(2)	4.21(1) × 10^–6^

a Moment for Eu^2+^ employed to calculate *x*.

### 3.2 Electronic Transport

**Figure 6:**
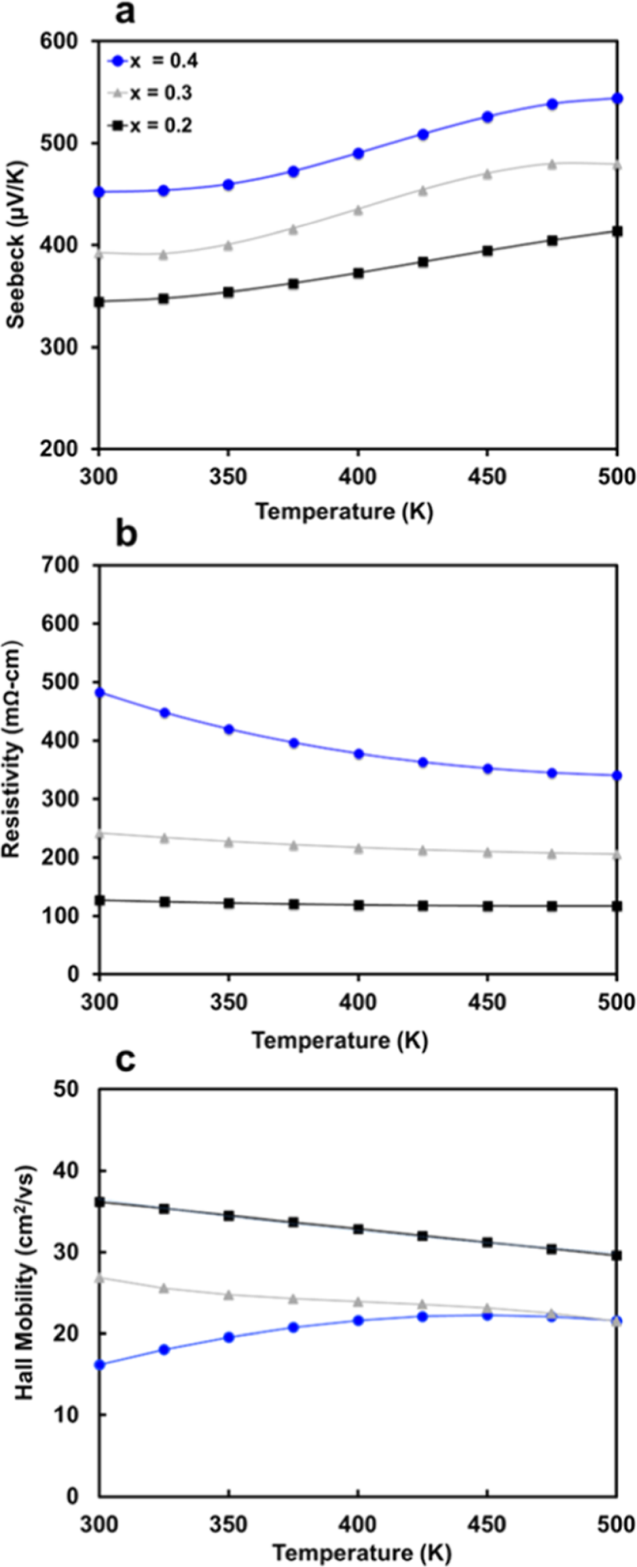
Polynomial fits of (a) Seebeck coefficients as a function of temperature; (b) electrical resistivities; and (c) Hall mobilities for Ba_2–*x*_Eu_*x*_ZnSb_2_ (*x* = 0.2, 0.3, and 0.4).

Figure 6 displays the Seebeck, resistivity, and Hall mobility as polynomial fits to the data from the heating cycle. Experimental data with the polynominal fit shown are presented in Supporting Information, Figures S4 and S5. Carrier concentrations of the samples are quite low, on the order of 10^17^–10^18^ carriers/cm^–3^ (Table 2), and decrease with increasing Eu content. Given that Eu is confirmed to be in the 2+ oxidation state via magnetism, the reduction in charge carriers may be due to the changing defect energies of Eu compared to Ba, as has been shown in AZn_2_Sb_2_ (A = Yb, Eu, Sr, and Ca), or to the higher electronegativity of Eu compared to Ba that could lead to incomplete electron donation.^30,31^ The Curie–Weiss behavior of the magnetism indicates the presence of the local magnetic moment from the Eu^2+^ that is substituted for Ba, which is consistent with the experimental nonmetallic resistivity expected due to the Zintl formalism. Seebeck coefficients (Figure 6a) are high (>300 μV/K) across the entire temperature range and increase with temperature, reaching a maximum value of ∼550 μV/K for *x* = 0.4 at 500 K. In their theoretical study on the electronic structure and thermoelectric properties of Ba_2_ZnSb_2_, Zhang et al.^18^ predicted values >350 μV/K for <10^19^ carriers/cm^–3^ at 600 K, and experimental values range from 350 to 450 μV/K at 500 K. The experimental Seebeck coefficients reported here agree reasonably well with those predicted from theory, although direct comparison is not possible for all compositions due to the low carrier concentrations in the synthesized samples and lower measurement temperatures.

**Table 2:** Hall Carrier Concentration at 300 K for Ba_2–*x*_Eu_*x*_ZnSb_2_

*x*	carrier concentration (cm^–3^), 300 K
0.2	1.41 × 10^18^
0.3	1.02 × 10^18^
0.4	6.5 × 10^17^

Figure 6b shows electrical resistivities that decrease with temperature as expected for a lightly doped semiconductor.^18^Figure 6c gives Hall mobilities, which are high for a Zintl phase (up to 36 cm^2^/V s) and consistent with expectations from the infinite tetrahedral chains.^2,3^ Electrical resistivity increases as more Eu is incorporated, consistent with the increase in Seebeck coefficient and decrease in carrier concentration. However, Hall mobility, which is expected to increase given a lower carrier concentration in a single parabolic band (SPB) model, where 1/ρ = *ne*μ also decreases with increasing Eu content. The deviation from SPB behavior is expected, given that band structures calculated for Ba_2_ZnSb_2_ are anisotropic with four degenerate bands present near the valence band edge. Experimentally determined effective masses also vary with carrier concentration (m* = 0.68, 1.07, and 1.19 *m*_e_ at 300 K for *x* = 0.2, 0.3, and 0.4, respectively), which can be an indication of anisotropy or multivalley transport.^32,33^ The valence band edge is composed of strongly hybridized Sb–Zn states with minimal contribution from Ba cations, while the conduction band has substantial Ba character.^5,18^ This suggests that substitution on the Ba site is unlikely to significantly alter bonding near the VBM, and changing mobility effective masses are likely a result of band anisotropy or the presence of additional light bands just below the Fermi level. As Eu content decreases, the Fermi level moves toward the valence band, and the two light bands are accessed while dispersion in the anisotropic heavier bands increases, resulting in a lighter effective mass with greater mobility.^5,18^

High charge carrier mobility that improves with doping makes this system promising for further optimization, and a high *zT* is predicted for Ba_2_ZnSb_2_ if the carrier concentration can be increased to ∼10^20^ h^+^/cm^3^. For *x* = 0.2 and 0.3, mobility exhibits negative temperature dependence, indicating that acoustic phonon scattering is the dominant mechanism, while *x* = 0.4 shows positive temperature dependence at low temperatures (<425 K). This suggests activated mobility, which may be caused by grain boundary resistance, and could be an indication that the solubility limit of Eu has been reached for *x* = 0.4, leaving a small amount of unreacted Eu present at the grain boundaries.^9−11,34^ This drop in low-temperature mobility explains the higher electrical resistivity for *x* = 0.4 compared to *x* = 0.2 and 0.3, as well as a sharper decrease from 300 to 425 K.

## 4 Thermal Transport

**Figure 7:**
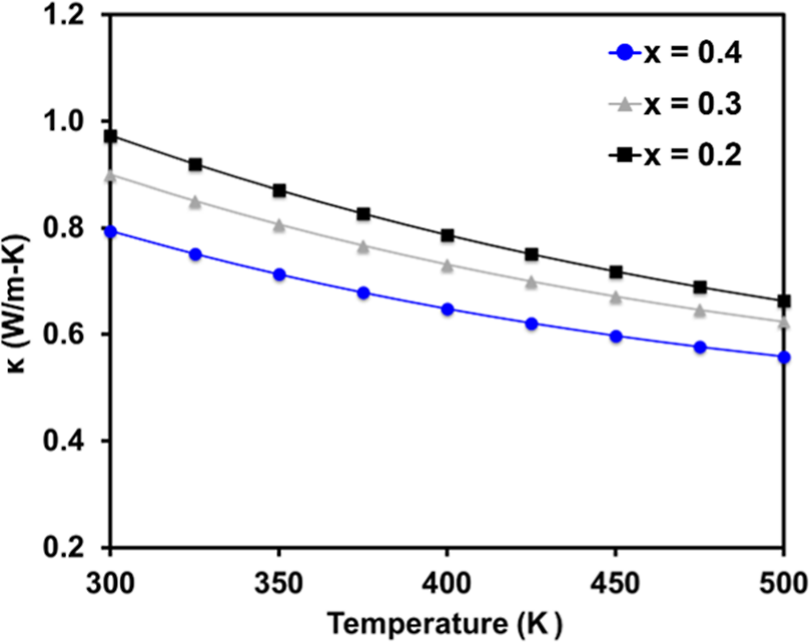
Polynomial fit of total thermal conductivity as a function of temperature for Ba_2–*x*_Eu_*x*_ZnSb_2_ [*x* = 0.2 (black-filled squares), 0.3 (gray-filled triangles), and 0.4 (blue-filled circles)].

Thermal conductivity calculated from thermal diffusivity and heat capacity (Dulong–Petit law) is shown in Figure 7. The Lorenz number was calculated from the Seebeck coefficient using the method described in ref (35) and the electronic component of the thermal conductivity determined via the Wiedemann–Franz relationship was negligible (<0.007 W/m K) due to the high electrical resistivity and low carrier concentration. Thus, the total thermal conductivity and the lattice component are virtually the same, and only the total thermal conductivity is shown. Thermal conductivity is low and comparable to the structurally similar Zintl phases Ca_5_M_2_Sb_6_ (M = Al, Ga, and In), Ca_3_AlSb_3_, and Sr_3_GaSb_3_, ranging from ∼1 to 0.55 W/m K. The drop in thermal conductivity with Eu incorporation can be attributed to alloy scattering due to the size difference between Ba and Eu, which is an effective method of reducing lattice thermal conductivity in other Zintl phases.^2,36^

### 4.1 Thermoelectric Figure of Merit (*zT*)

**Figure 8:**
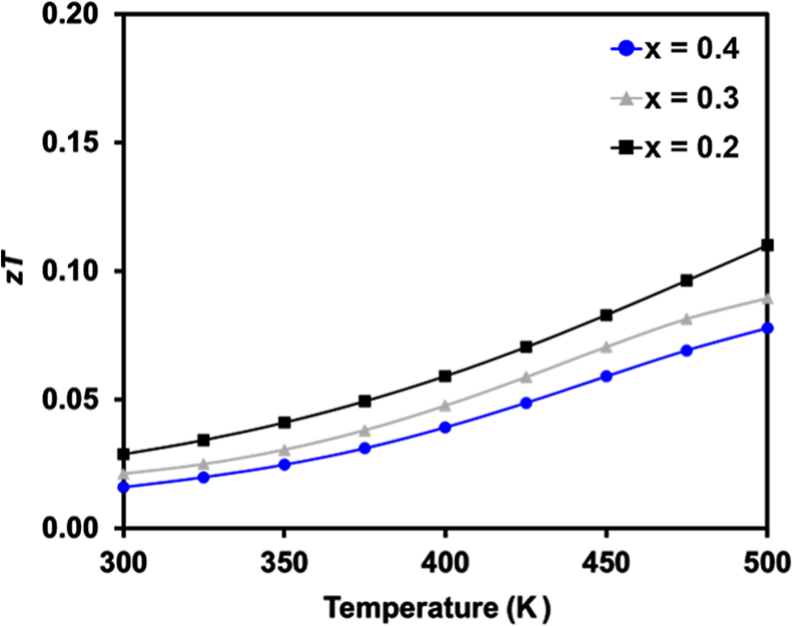
Temperature-dependent *zT*’s for Ba_2–*x*_Eu_*x*_ZnSb_2_ [*x* = 0.2 (black-filled squares), 0.3 (gray-filled triangles), and 0.4 (blue-filled circles)].

Calculated *zT*s as a function of temperature for Ba_2–*x*_Eu_*x*_ZnSb_2_ (*x* = 0.2, 0.3, and 0.4) are given in Figure 8. A maximum *zT* of 0.11 is obtained at 500 K for *x* = 0.2, the sample with the highest carrier concentration. This value is lower than the predicted maximum *zT* ≈ 0.8 at 600 K with 10^20^ carriers/cm^3^ for Ba_2_ZnSb_2_ because of the low carrier concentration in the synthesized samples, as well as the higher lattice thermal conductivity, 0.65 W/m K compared to the theoretically predicted values of κ_min_ = 0.41 and 0.11 W/m K.^18,20^ It may be possible to obtain an experimental *zT* closer to the predicted value if carrier concentrations can be increased. This may be possible via K-doping on the Ba site. Incorporating less air-sensitive elements may also improve *zT* by increasing the maximum operating temperature.

## 5 Conclusions

We have investigated thermoelectric properties for Ba_2–*x*_Eu_*x*_ZnSb_2_ (*x* = 0.2, 0.3, and 0.4), a Zintl phase with isolated infinite 1D chains of edge-shared [ZnSb_4/2_]^4–^ tetrahedra that give rise to anisotropic charge transport. A high *zT* was predicted for Ba_2_ZnSb_2_ due to the presence of four degenerate bands (two heavy and two light) just below the Fermi level. Experimental data confirm a high Seebeck coefficient and charge carrier mobility, as well as low lattice thermal conductivity. Predicted *zT*s are higher than experimental values due to low carrier concentrations in synthesized samples but could likely be improved by aliovalent substitution as the carrier concentrations are quite low for these Ba_2–*x*_Eu_*x*_ZnSb_2_ compounds. The air sensitivity of the samples limited the measurement temperature range, and further improvements to *zT* could likely be made by substituting with more air-stable elements to allow measurement to higher temperatures. Since the solubility range of Eu is limited, another potential route for optimization is substituting Na^1+^ for some Eu^2+^ in this Ba/Eu solid solution or combining both La^3+^ and K^+^ for Ba to synthesize LaKZnSb_2_, a hypothetical charge-balanced compound that may be expected to retain the same structure type due to the similar average size of La/K and Ba and is expected to be less air-sensitive than Ba_2_ZnSb_2_ due to the greater electronegativity of La compared to Ba. Substituting Cu for Zn or Sn for Sb in Ba_2–*x*_Eu_*x*_ZnSb_2_ is another possible route for optimization.

## Supporting Information Available

Rietveld refinements of A_11_Sb_10_ (A = Ba and Eu); Rietveld refinement parameters for A_11_Sb_10_ (A = Ba and Eu); Rietveld refinements of Ba_2–*x*_Eu_*x*_ZnSb_2_; Rietveld refinement parameters for Ba_2–*x*_Eu_*x*_ZnSb_2_; experimental Seebeck and electronic transport data from the first heating cycle; thermal conductivity data from the first cycle; SEM micrographs; a typical EDS elemental map; plot of magnetization vs T for the three samples; and inverse magnetic susceptibility with Curie–Weiss fits and parameters (PDF).

## References

[ref1] Ovchinnikov A.; Bobev S. Zintl Phases with Group 15 Elements and the Transition Metals: A Brief Overview of Pnictides with Diverse and Complex Structures. J. Solid State Chem. 2019, 270, 346–359. 10.1016/j.jssc.2018.11.029.

[ref2] Snyder G. J.; Toberer E. S. Complex Thermoelectric Materials. Nat. Mater. 2010, 7, 101–110. 10.1142/9789814317665_0016. 18219332

[ref3] Shuai J.; Mao J.; Song S.; Zhang Q.; Chen G.; Ren Z. Recent Progress and Future Challenges on Thermoelectric Zintl Materials. Mater. Today Phys. 2017, 1, 74–95. 10.1016/J.MTPHYS.2017.06.003.

[ref4] Zevalkink A.; Smiadak D. M.; Blackburn J. L.; Ferguson A. J.; Chabinyc M. L.; Delaire O.; Wang J.; Kovnir K.; Martin J.; Schelhas L. T.; Sparks T. D.; Kang S. D.; Dylla M. T.; Snyder G. J.; Ortiz B. R.; Toberer E. S. A Practical Field Guide to Thermoelectrics: Fundamentals, Synthesis, and Characterization. Appl. Phys. Rev. 2018, 5, 021303 10.1063/1.5021094.

[ref5] Sun J.; Singh D. J. Thermoelectric Properties of AMg_2_X_2_, AZn_2_Sb_2_ (A = Ca, Sr, Ba; X = Sb, Bi), and Ba_2_ZnX_2_ (X = Sb, Bi) Zintl Compounds. J. Mater. Chem. A 2017, 5, 8499–8509. 10.1039/c6ta11234j.

[ref6] Parker D.; Chen X.; Singh D. J. High Three-Dimensional Thermoelectric Performance from Low-Dimensional Bands. Phys. Rev. Lett. 2013, 110, 146601–146605. 10.1103/PhysRevLett.110.146601. 25167018

[ref7] Dylla M. T.; Kang S. D.; Snyder G. J. Effect of Two-Dimensional Crystal Orbitals on Fermi Surfaces and Electron Transport in Three-Dimensional Perovskite Oxides. Angew. Chem., Int. Ed. 2019, 58, 5503–5512. 10.1002/anie.201812230. 30589168

[ref8] Dresselhaus M. S.; Chen G.; Tang M. Y.; Yang R.; Lee H.; Wang D.; Ren Z.; Fleurial J.-P.; Gogna P. New Directions for Low-Dimensional Thermoelectric Materials. Adv. Mater. 2007, 19, 1043–1053. 10.1002/adma.200600527.

[ref9] Zevalkink A.; Toberer E. S.; Zeier W. G.; Flage-Larsen E.; Snyder G. J. Ca_3_AlSb_3_: An Inexpensive, Non-Toxic Thermoelectric Material for Waste Heat Recovery. Energy Environ. Sci. 2011, 4, 510–518. 10.1039/c0ee00517g.

[ref10] Zevalkink A.; Zeier W. G.; Pomrehn G.; Schechtel E.; Tremel W.; Snyder G. J. Thermoelectric Properties of Sr_3_GaSb_3_-a Chain-Forming Zintl Compound. Energy Environ. Sci. 2012, 5, 9121–9128. 10.1039/c2ee22378c.

[ref11] Zevalkink A.; Pomrehn G. S.; Johnson S.; Swallow J.; Gibbs Z. M.; Snyder G. J. Influence of the Triel Elements (M = Al, Ga, In) on the Transport Properties of Ca_5_M_2_Sb_6_ Zintl Compounds. Chem. Mater. 2012, 24, 2091–2098. 10.1021/cm300520w.

[ref12] Chanakian S.; Uhl D.; Neff D.; Drymiotis F.; Park J.; Petkov V.; Zevalkink A.; Bux S. Exceptionally High Electronic Mobility in Defect-Rich Eu_2_ZnSb_2-x_Bi_x_ Alloys. J. Mater. Chem. A 2020, 8, 6004–6012. 10.1039/c9ta14170g.

[ref13] Cooley J. A.; Promkhan P.; Gangopadhyay S.; Donadio D.; Pickett W. E.; Ortiz B. R.; Toberer E. S.; Kauzlarich S. M. High Seebeck Coefficient and Unusually Low Thermal Conductivity Near Ambient Temperatures in Layered Compound Yb_2-x_Eu_x_CdSb_2_. Chem. Mater. 2018, 30, 484–493. 10.1021/acs.chemmater.7b04517.

[ref14] Devlin K. P.; Chen S.; Donadio D.; Kauzlarich S. M. Solid Solution Yb_2-x_Ca_x_CdSb_2_: Structure, Thermoelectric Properties, and Quality Factor. Inorg. Chem. 2021, 60, 13596–13606. 10.1021/acs.inorgchem.1c01906. 34415765

[ref15] Chen C.; Xue W.; Li S.; Zhang Z.; Li X.; Wang X.; Liu Y.; Sui J.; Liu X.; Cao F.; Ren Z.; Chu C.-W.; Wang Y.; Zhang Q. Zintl-Phase Eu_2_ZnSb_2_: A Promising Thermoelectric Material with Ultralow Thermal Conductivity. Proc. Natl. Acad. Sci. 2019, 116, 2831–2836. 10.1073/PNAS.1819157116. 30718395 PMC6386660

[ref16] Chen C.; Li X.; Xue W.; Bai F.; Huang Y.; Yao H.; Li S.; Zhang Z.; Wang X.; Sui J.; Liu X.; Cao F.; Wang Y.; Zhang Q. Manipulating the Intrinsic Vacancies for Enhanced Thermoelectric Performance in Eu_2_ZnSb_2_ Zintl Phase. Nano Energy 2020, 73, 104771–104778. 10.1016/j.nanoen.2020.104771.

[ref17] Shi Q.; Feng Z.; Yan Y.; Wang Y. X. Electronic Structure and Thermoelectric Properties of Zintl Compounds A_3_AlSb_3_ (A = Ca and Sr): First-Principles Study. RSC Adv. 2015, 5, 65133–65138. 10.1039/c5ra09804a.

[ref18] Zhang X.; Wang C.; Wang Y. X. Influence of the Elements (Pn = As, Sb, Bi) on the Transport Properties of p-Type Zintl Compounds Ba_2_ZnPn_2_. Comput. Mater. Sci. 2017, 127, 8–14. 10.1016/j.commatsci.2016.10.022.

[ref19] Saparov B.; Bobev S. Isolated ^1^_∞_[ZnPn_2_]^4-^ Chains in the Zintl Phases Ba_2_ZnPn_2_ (Pn = As, Sb, Bi)--Synthesis, Structure, and Bonding. Inorg. Chem. 2010, 49, 5173–5179. 10.1021/ic100296x. 20426404

[ref20] Zhai W.; Li L.; Zhao M.; Hu Q.; Li J.; Yang G.; Yan Y.; Zhang C.; Liu P. F. Phonon Transport in Zintl Ba_2_ZnAs_2_ and Ba_2_ZnSb_2_: A First-Principles Study. Mater. Sci. Semicond. Process. 2022, 141, 106446 10.1016/j.mssp.2021.106446.

[ref21] Justl A. P.; Cerretti G.; Bux S. K.; Kauzlarich S. M. 2 + 2 = 3: Making Ternary Phases through a Binary Approach. Chem. Mater. 2022, 34, 1342–1355. 10.1021/acs.chemmater.1c04031.

[ref22] Iwanaga S.; Toberer E. S.; Lalonde A.; Snyder G. J. A High Temperature Apparatus for Measurement of the Seebeck Coefficient. Rev. Sci. Instrum. 2011, 82, 063905 10.1063/1.3601358. 21721707

[ref23] Borup K. A.; Toberer E. S.; Zoltan L. D.; Nakatsukasa G.; Errico M.; Fleurial J.-P.; Iversen B. B.; Snyder G. J. Measurement of the Electrical Resistivity and Hall Coefficient at High Temperatures. Rev. Sci. Instrum. 2012, 83, 123902 10.1063/1.4770124. 23278000

[ref24] Wilson D. K.; Saparov B.; Bobev S. S. Crystal Structures and Properties of the Zintl Phases Sr_2_ZnP_2_, Sr_2_ZnAs_2_, A_2_ZnSb_2_ and A_2_ZnBi_2_ (A = Sr and Eu). Z. fur Anorg. Allg. Chem. 2011, 637, 2018–2025. 10.1002/zaac.201100177.

[ref25] Ovchinnikov A.; Darone G. M.; Saparov B.; Bobev S. Exploratory Work in the Quaternary System of Ca-Eu-Cd-Sb: Synthesis, Crystal, and Electronic Structures of New Zintl Solid Solutions. Materials 2018, 11, 2146–2213. 10.3390/ma11112146. 30384471 PMC6265713

[ref26] Saparov B.; Saito M.; Bobev S. Syntheses, and Crystal and Electronic Structures of the New Zintl Phases Na_2_ACdSb_2_ and K_2_ACdSb_2_ (A=Ca, Sr, Ba, Eu, Yb): Structural Relationship with Yb_2_CdSb_2_ and the Solid Solutions Sr_2–x_*A*_x_CdSb_2_, Ba_2–x_*A*_x_CdSb_2_ and Eu_2–x_Yb_x_CdSb_2_. J. Solid State Chem. 2011, 184, 432–440. 10.1016/j.jssc.2010.12.015.

[ref27] Radzieowski M.; Stegemann F.; Klenner S.; Zhang Y.; Fokwa B. P. T.; Janka O. On the Divalent Character of the Eu Atoms in the Ternary Zintl Phases Eu_5_In_2_Pn_6_ and Eu_3_MAs_3_ (Pn = As-Bi; M = Al, Ga). Mater. Chem. Front. 2020, 4, 1231–1248. 10.1039/c9qm00703b.

[ref28] Radzieowski M.; Block T.; Fickenscher T.; Zhang Y.; Fokwa B. P. T.; Janka O. S. Crystal and Electronic Structures, Physical Properties and ^121^Sb and ^151^Eu Mössbauer Spectroscopy of the Alumo-Antimonide Zintl-Phase Eu_5_Al_2_Sb_6_. Mater. Chem. Front. 2017, 1, 1563–1572. 10.1039/c7qm00057j.

[ref29] Radzieowski M.; Block T.; Klenner S.; Zhang Y.; Fokwa B. P. T.; Janka O. S. Crystal and Electronic Structure, Physical Properties and ^121^Sb and ^151^Eu Mössbauer Spectroscopy of the Eu_14_AlPn_11_ Series (Pn = As, Sb). Inorg. Chem. Front. 2019, 6, 137–147. 10.1039/c8qi01099d.

[ref30] Pomrehn G. S.; Zevalkink A.; Zeier W. G.; Van De Walle A.; Snyder G. J. Defect-Controlled Electronic Properties in AZn_2_Sb_2_ Zintl Phases. Angew. Chem., Int. Ed. 2014, 53, 3422–3426. 10.1002/anie.201311125. 24616066

[ref31] Shuai J.; Wang Y.; Liu Z.; Kim H. S.; Mao J.; Sui J.; Ren Z. Enhancement of Thermoelectric Performance of Phase Pure Zintl Compounds Ca_1-x_Yb_x_Zn_2_Sb_2_, Ca_1-x_Eu_x_Zn_2_Sb_2_, and Eu_1-x_Yb_x_Zn_2_Sb_2_. Nano Energy 2016, 25, 136–144. 10.1016/J.NANOEN.2016.04.023.

[ref32] Perez C. J.; Wood M.; Ricci F.; Yu G.; Vo T.; Bux S. K.; Hautier G.; Rignanese G.-M.; Snyder G. J.; Kauzlarich S. M. Discovery of Multivalley Fermi Surface Responsible for the High Thermoelectric Performance in Yb_14_MnSb_11_ and Yb_14_MgSb_11_. Sci. Adv. 2021, 7, eabe9439 10.1126/sciadv.abe9439. 33523935 PMC7817104

[ref33] Kang S. D.; Snyder G. J. Transport Property Analysis Method for Thermoelectric Materials: Material Quality Factor and the Effective Mass Model. Advances in Thermoelectricity: Foundational Issues, Materials and Nanotechnology; IOS Press, 2017; pp 27–36.

[ref34] Kuo J. J.; Kang S. D.; Imasato K.; Tamaki H.; Ohno S.; Kanno T.; Snyder G. J. Grain Boundary Dominated Charge Transport in Mg_3_Sb_2_-Based Compounds. Energy Environ. Sci. 2018, 11, 429–434. 10.1039/c7ee03326e.

[ref35] Kim H. S.; Gibbs Z. M.; Tang Y.; Wang H.; Snyder G. J. Characterization of Lorenz Number with Seebeck Coefficient Measurement. APL Mater. 2015, 3, 041506 10.1063/1.4908244.

[ref36] Liu K. F.; Xia S. Q. Recent Progresses on Thermoelectric Zintl Phases: Structures, Materials and Optimization. J. Solid State Chem. 2019, 270, 252–264. 10.1016/j.jssc.2018.11.030.

