# Supplementary material for: Thermoelectric Properties of Ba2–xEuxZnSb2, a Zintl Phase with One-Dimensional Covalent Chains
Source: Inorg Chem. 2023 Apr 6;62(15):6003–10. doi: 10.1021/acs.inorgchem.2c04484 (PMC10114066; doi:10.1021/acs.inorgchem.2c04484)
Supplement: Supplementary file 1 — ic2c04484_si_001.pdf [file ic2c04484_si_001.pdf]

Thermoelectric properties of $Ba_{2-x}Eu_xZnSb_2$, a Zintl phase with one-dimensional covalent chains

Ashlee K. Hauble,[1] Kamil Ciesielski,[2] Valentin Taufour,[3] Eric S. Toberer,[2] and Susan M. Kauzlarich[1]

Affiliations

[1]Department of Chemistry, University of California, One Shields Ave, Davis, California 95616, United States

[2]Department of Physics, Colorado School of Mines, 1500 Illinois St, Golden, CO 80401, United States

[3]Department of Physics and Astronomy, University of California, One Shields Avenue, Davis, California 95616, United States

*Corresponding author's emails: smkauzlarich@ucdavis.edu

Supporting Information

Table of Contents

Figure S1. Rietveld refinements of PXRD data of $Ba_{11}Sb_{10}$ and $Eu_{11}Sb_{10}$.

Table S1. Rietveld Refinement Parameters from PXRD for $Ba_{11}Sb_{10}$ and $Eu_{11}Sb_{10}$

Figure S2-S3. Rietveld refinements of PXRD data of $Ba_{2-x}Eu_xZnSb_2$ (x = 0, 0.2, 0.3, 0.4).

Table S2. Rietveld Refinements Parameters from PXRD for $Ba_{2-x}Eu_xZnSb_2$ (x = 0, 0.2, 0.3, 0.4)

Figure S4. Seebeck and electronic data (points from the first heating cycle with polynominal fits shown as lines

Figure S5. Thermal conductivity data (points from the first heating cycle with polynominal fits shown as lines

Figure S6. SEM micrographs and EDS elemental maps

Figure S7. EDS spectrum

Figure S8. Magnetic susceptibility of the three samples on one graph.

Figure S9. Inverse magnetic susceptibility with the fits to the data.

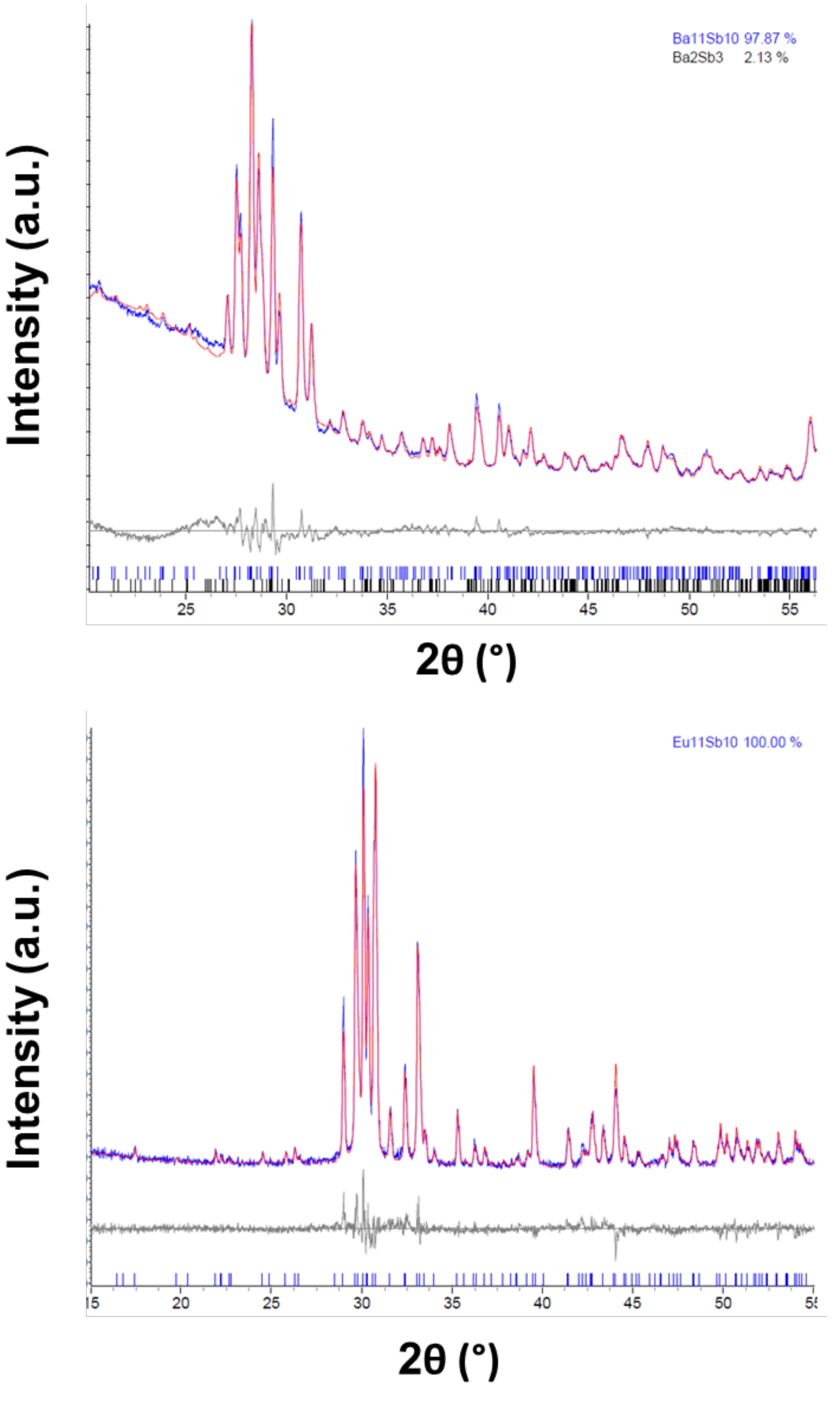

Figure S1. Rietveld refinements of $Ba_{11}Sb_{10}$ (top) and $Eu_{11}Sb_{10}$ (bottom). The observed data are shown in black, calculated pattern in red, and difference curve is in gray. The sloping background observed at low angle is due to scattering from the Kapton tape used to keep the sample air free. Bragg peaks are indicated by tick marks at the bottom with the legend listed in each plot.

Table S1. Selected Rietveld Refinement Parameters and Statistics for $Ba_{11}Sb_{10}$ and $Eu_{11}Sb_{10}$

**x**		**$Ba_{11}Sb_{10}$**	**$Eu_{11}Sb_{10}$**
**Unit Cell Parameters**	*a* (Å)	12.6953(6)	12.3338(6)
	c (Å)	13.2059(7)	17.9957(9)
	V (Å^3)	3269.8(3)	2737.5(3)
**Rp (%), Rwp (%)**		3.07, 3.89	8.86, 11.48
**$A_{11}Sb_{10}$ (wt %)**		97.87	100
**Ba_2Sb_3 (wt %)**		2.13	0

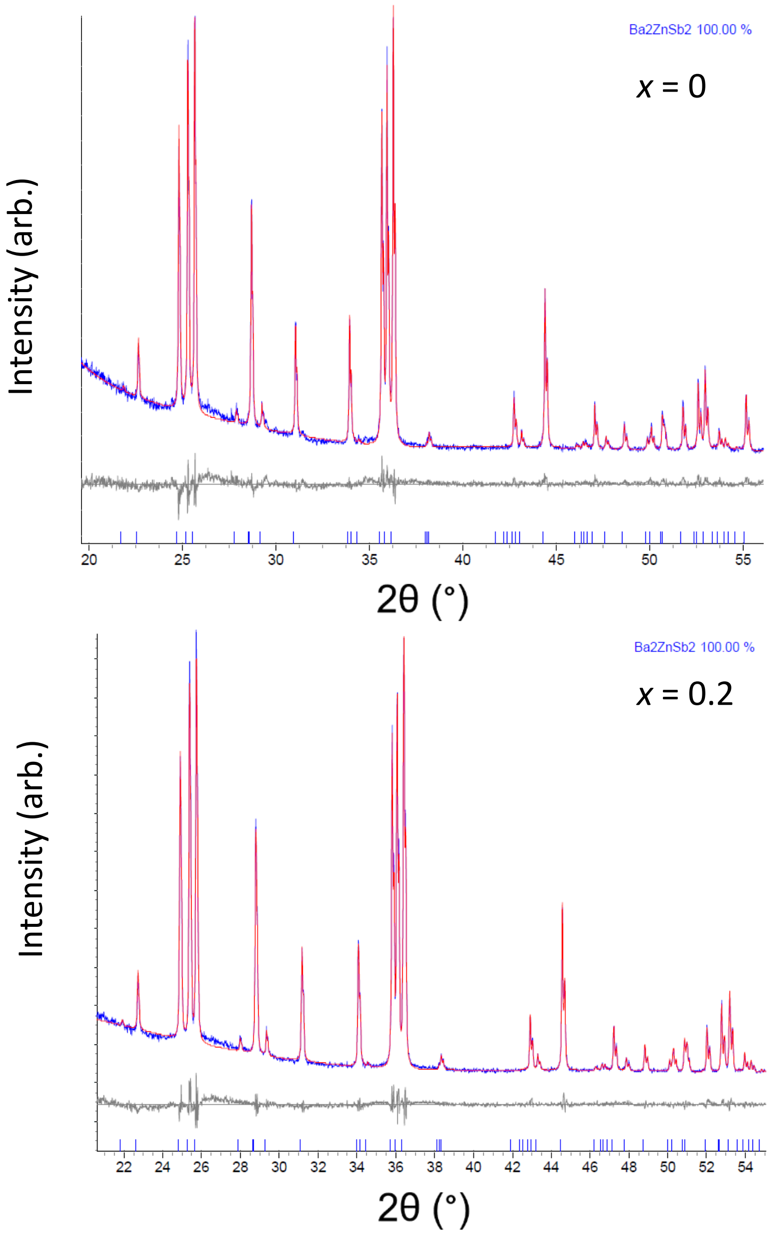

Figure S2. Rietveld refinements of $Ba_{2-x}Eu_xZnSb_2$ (x = 0, 0.2). The observed data are shown in black, calculated pattern in red, and difference curve is in gray. The sloping background observed at low angle is due to scattering from the Kapton tape used to keep the sample air free. Bragg peaks are indicated by tick marks at the bottom with the legend listed in each plot.

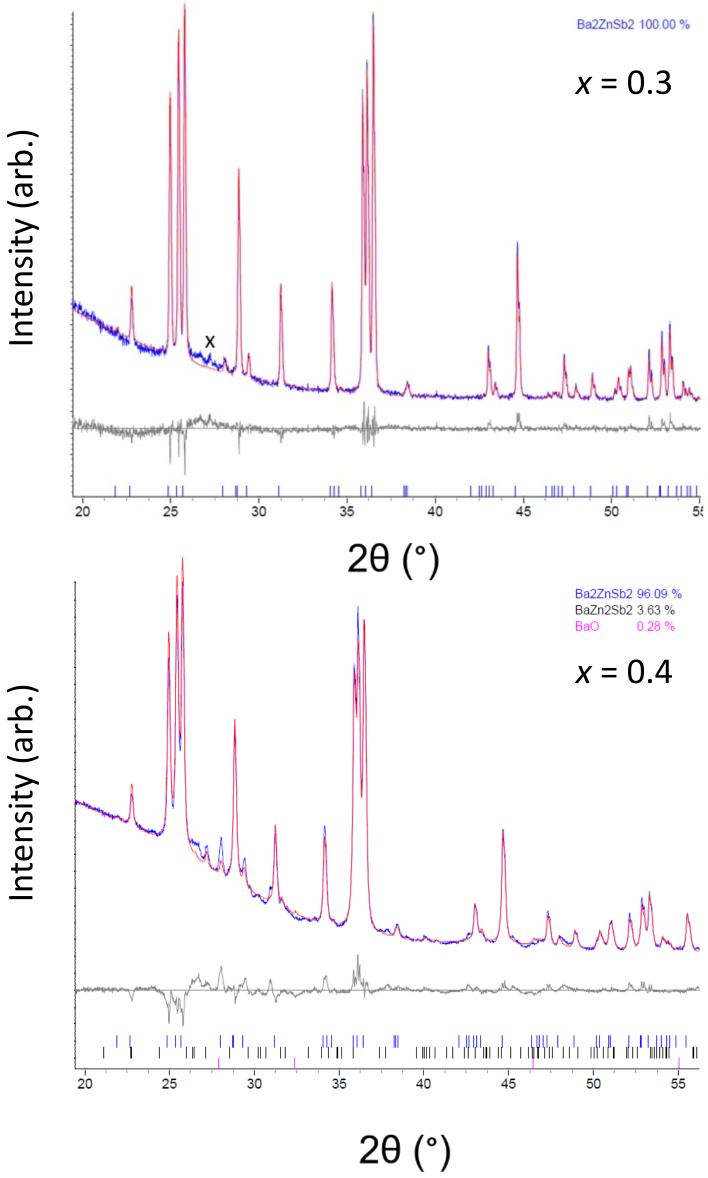

Figure S3. Rietveld refinements of $Ba_{2-x}Eu_xZnSb_2$ (x = 0.3, 0.4). The observed data are shown in black, calculated pattern in red, and difference curve is in gray. The sloping background observed at low angle is due to scattering from the Kapton tape used to keep the sample air free. The x marks an unidentified impurity peak. Bragg peaks are indicated by tick marks at the bottom with the legend listed in each plot.

Table S2. Selected Rietveld Refinement Parameters and Statistics of $Ba_{2-x}Eu_xZnSb_2$ (x = 0, 0.2, 0.3, 0.4)

<table>
<tr><th>x</th><th></th><th>0</th><th colspan="2">0.2</th><th>0.3</th><th>0.4</th></tr>
<tr><td rowspan="4">Unit Cell Parameters</td><td>a (Å)</td><td>14.1400(2)</td><td colspan="2">14.0805(3)</td><td>14.0490(4)</td><td>14.0374(3)</td></tr>
<tr><td>b (Å)</td><td>7.2041(1)</td><td colspan="2">7.175(1)</td><td>7.1599(2)</td><td>7.1541(2)</td></tr>
<tr><td>c (Å)</td><td>6.9681(1)</td><td colspan="2">6.946(1)</td><td>6.9339(2)</td><td>6.9291(2)</td></tr>
<tr><td>V ($Å^3$)</td><td>709.81(2)</td><td colspan="2">701.76(2)</td><td>697.48(3)</td><td>695.86(3)</td></tr>
<tr><td>Rp (%), Rwp (%)</td><td></td><td>7.20, 9.19</td><td colspan="2">5.38, 6.96</td><td>5.95, 7.58</td><td>4.05, 5.20</td></tr>
<tr><td colspan="2">$Ba_{2-x}Eu_xZnSb_2$ (wt %)</td><td>-</td><td>100</td><td>100</td><td>100</td><td>95.76</td></tr>
<tr><td colspan="2">$BaZn_2Sb_2$ (wt %)</td><td>-</td><td>0</td><td>0</td><td>0</td><td>3.62</td></tr>
<tr><td colspan="2">BaO</td><td>0</td><td>0</td><td>0</td><td>0</td><td>0.62</td></tr>
</table>

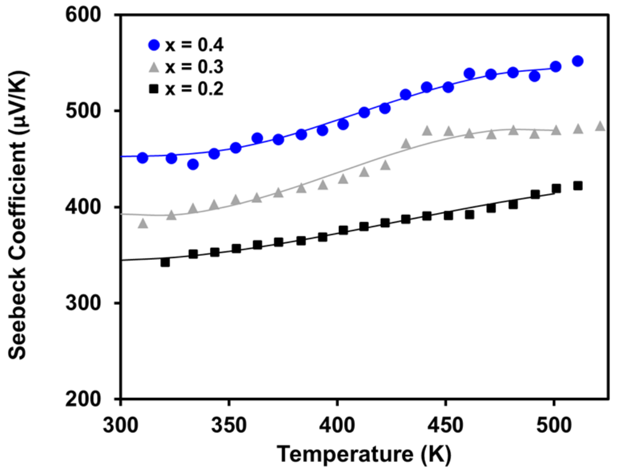

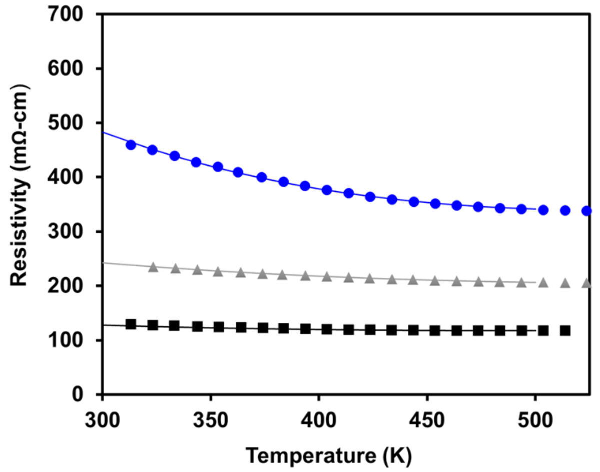

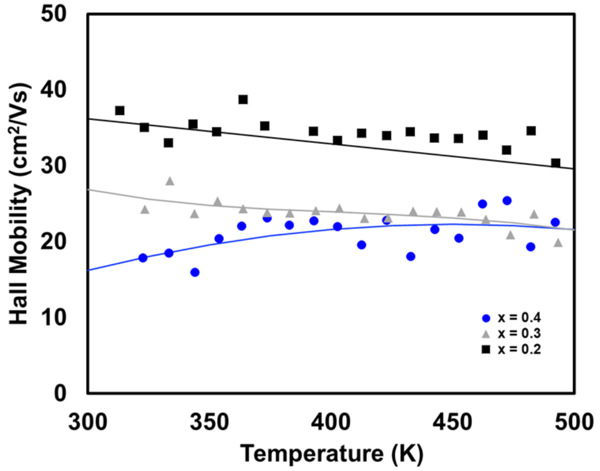

Figure S4. Electronic transport data (points) from the first heating cycle with lines showing the polynominal fits (lines).

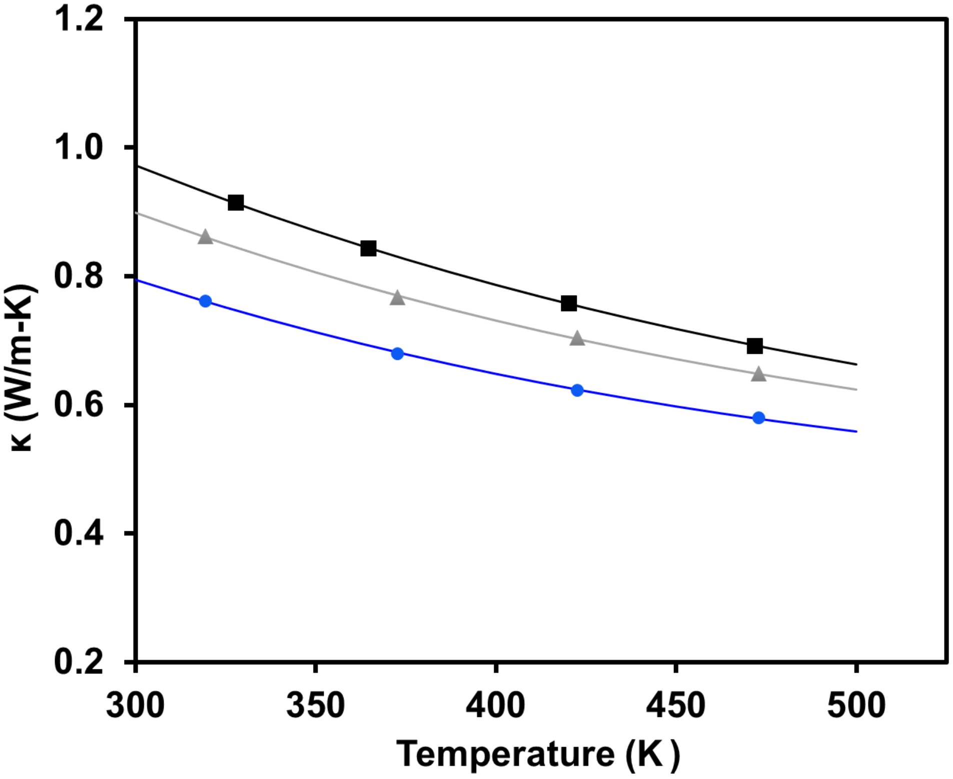

Figure S5. Thermal conductivity (points) from the first heating cycle with lines showing the polynominal fits (lines).

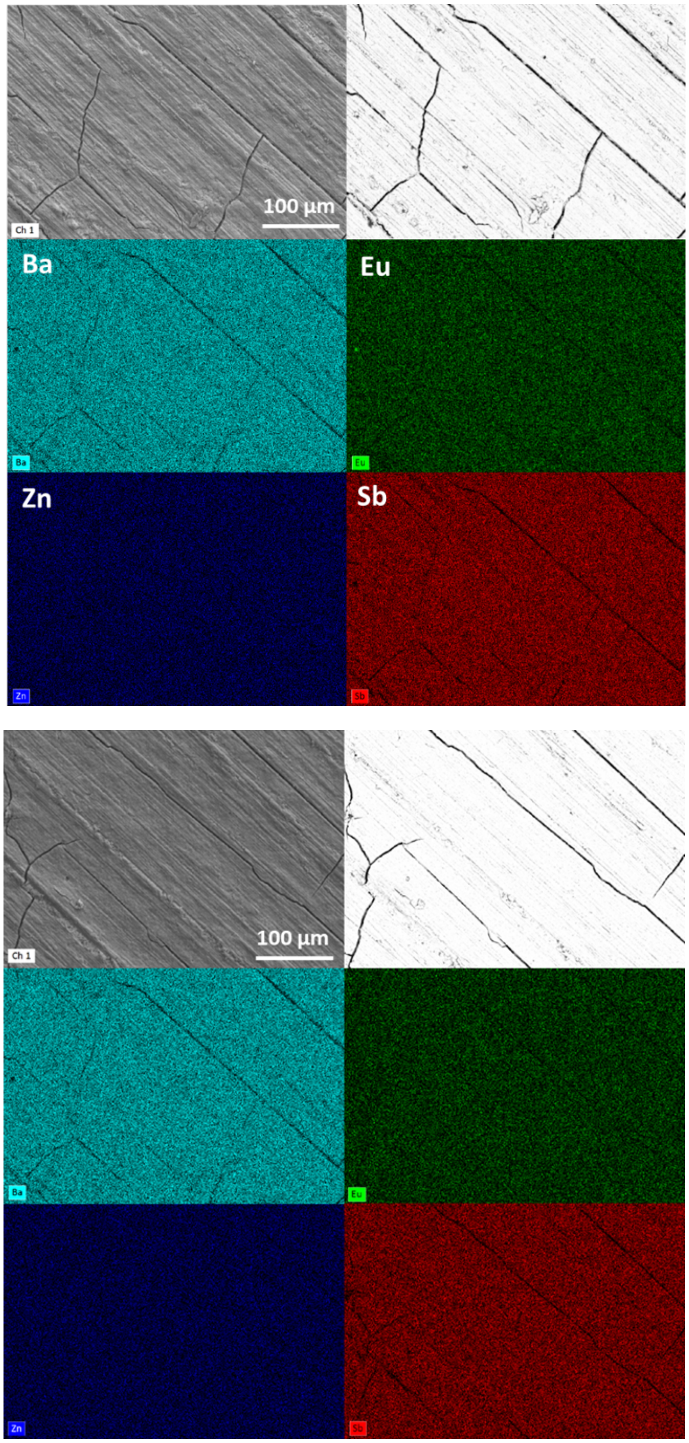

Figure S6. SEM micrographs and EDS elemental maps for $Ba_{1.8}Eu_{0.2}ZnSb_2$ (top) and $Ba_{1.7}Eu_{0.3}ZnSb_2$ (bottom).

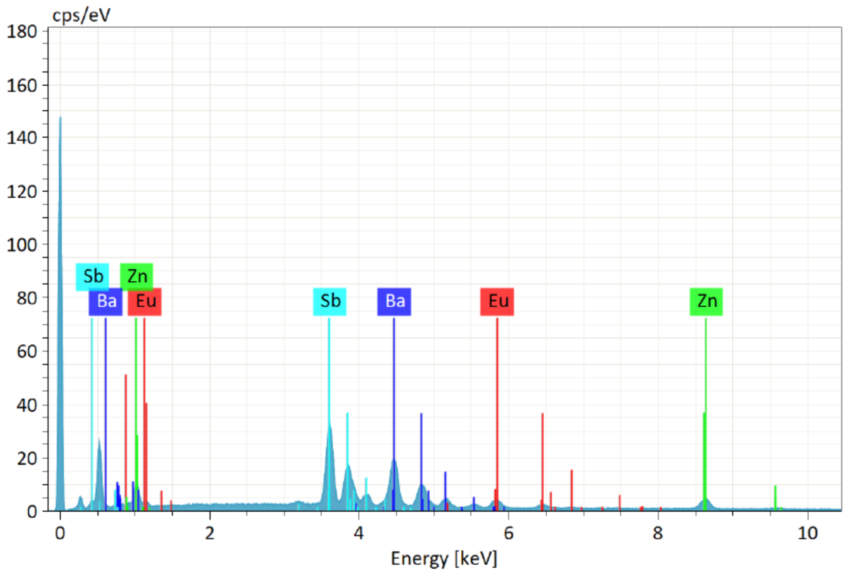

Figure S7. A Typical EDS spectrum for $Ba_{2-x}Eu_xZnSb_2$ samples.

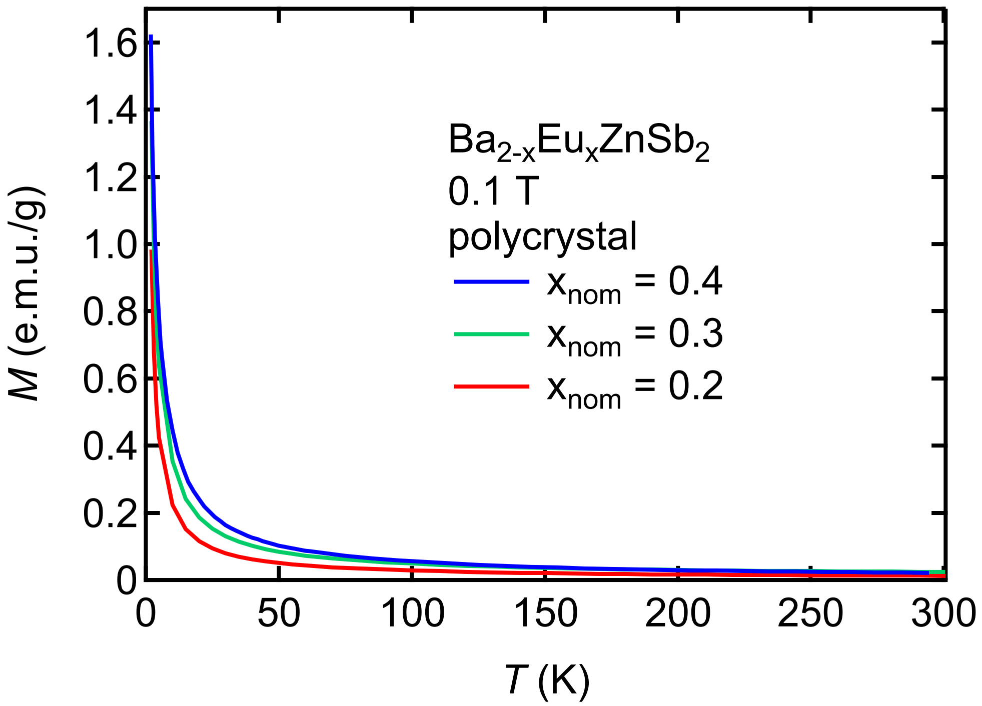

Figure S8. Magnetic susceptibility of the samples as a function of temperature.

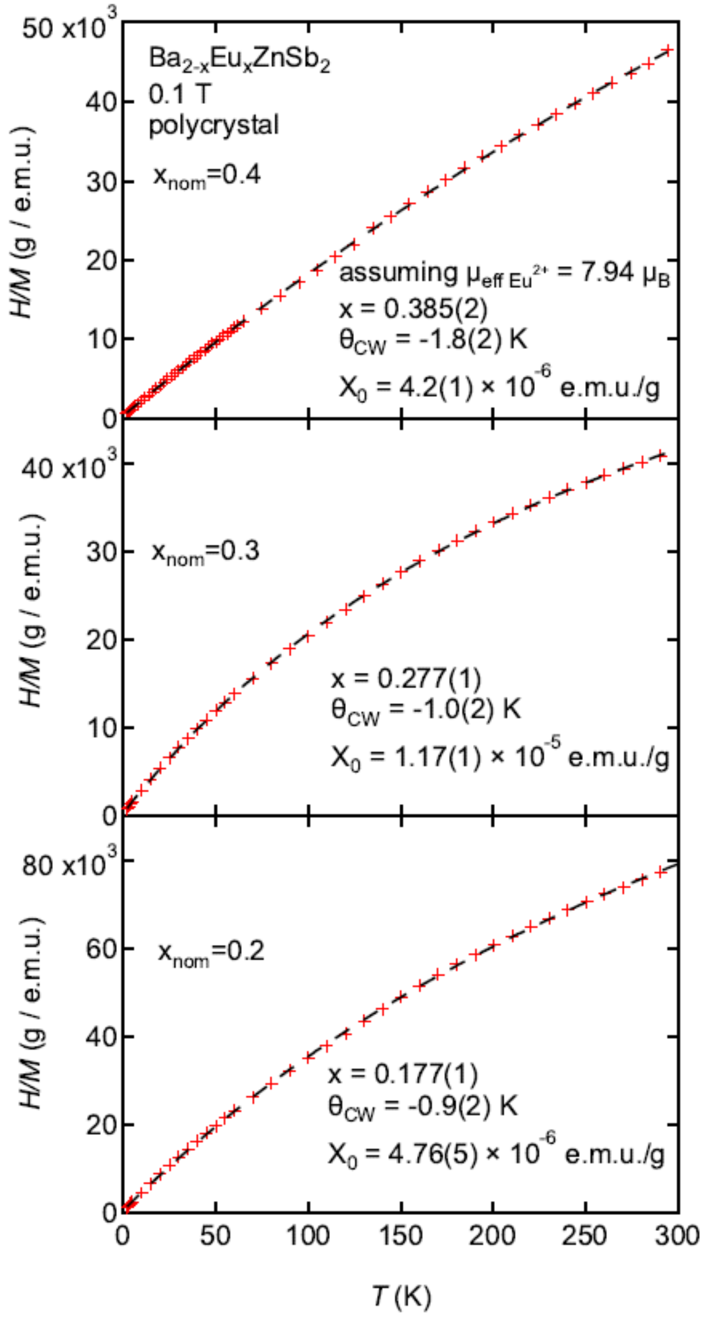

Figure S9. Inverse magnetic susceptibility (red) and modified Curie-Weiss fit (black).
